# Difference in Perseverative Errors during a Visual Attention Task with Auditory Distractors in Alpha-9 Nicotinic Receptor Subunit Wild Type and Knock-Out Mice

**DOI:** 10.3389/fncel.2017.00357

**Published:** 2017-11-08

**Authors:** Pascal Jorratt, Paul H. Delano, Carolina Delgado, Alexies Dagnino-Subiabre, Gonzalo Terreros

**Affiliations:** ^1^Departamento de Neurociencia, Facultad de Medicina, Universidad de Chile, Santiago, Chile; ^2^Departamento de Otorrinolaringología, Hospital Clínico de la Universidad de Chile, Santiago, Chile; ^3^Departamento Neurología y Neurocirugía, Hospital Clínico de la Universidad de Chile, Santiago, Chile; ^4^Laboratorio de Neurobiología del Stress, Centro de Neurobiología y Plasticidad Cerebral (CNPC), Instituto de Fisiología, Facultad de Ciencias, Universidad de Valparaíso, Valparaíso, Chile; ^5^Instituto de Ciencias de la Salud, Universidad de O’Higgins, Rancagua, Chile

**Keywords:** nicotinic receptors, olivocochlear, auditory efferent, selective attention, impulsivity

## Abstract

The auditory efferent system is a neural network that originates in the auditory cortex and projects to the cochlear receptor through olivocochlear (OC) neurons. Medial OC neurons make cholinergic synapses with outer hair cells (OHCs) through nicotinic receptors constituted by α9 and α10 subunits. One of the physiological functions of the α9 nicotinic receptor subunit (α9-nAChR) is the suppression of auditory distractors during selective attention to visual stimuli. In a recent study we demonstrated that the behavioral performance of alpha-9 nicotinic receptor knock-out (KO) mice is altered during selective attention to visual stimuli with auditory distractors since they made less correct responses and more omissions than wild type (WT) mice. As the inhibition of the behavioral responses to irrelevant stimuli is an important mechanism of the selective attention processes, behavioral errors are relevant measures that can reflect altered inhibitory control. Errors produced during a cued attention task can be classified as premature, target and perseverative errors. Perseverative responses can be considered as an inability to inhibit the repetition of an action already planned, while premature responses can be considered as an index of the ability to wait or retain an action. Here, we studied premature, target and perseverative errors during a visual attention task with auditory distractors in WT and KO mice. We found that α9-KO mice make fewer perseverative errors with longer latencies than WT mice in the presence of auditory distractors. In addition, although we found no significant difference in the number of target error between genotypes, KO mice made more short-latency target errors than WT mice during the presentation of auditory distractors. The fewer perseverative error made by α9-KO mice could be explained by a reduced motivation for reward and an increased impulsivity during decision making with auditory distraction in KO mice.

## Introduction

The auditory efferent system is a neural network that comprises descending projections from the auditory cortex to several subcortical nuclei, including the medial geniculate body, inferior colliculus, cochlear nucleus and superior olivary complex (Terreros and Delano, 2015). Corticofugal projections are also connected to the amygdala complex (Bose et al., 2010), and to the cochlear receptor through olivocochlear (OC) neurons (Mulders and Robertson, 2000), which are originated in the medial and lateral region of the superior olivary complex, constituting medial OC (MOC) and lateral OC (LOC) systems (Warr and Guinan, 1979). MOC neurons make cholinergic synapses with outer hair cells (OHCs) through nicotinic receptors constituted by α9 and α10 subunits that mediate auditory efferent activity (Elgoyhen et al., 1994, 2001, 2009). The α9 subunit of nicotinic receptors is expressed in different parts of the nervous and endocrine systems, including the inner ear, pituitary gland and dorsal ganglion root neurons (McIntosh et al., 2009). This subunit has been implicated in different physiological functions, including auditory frequency discrimination (Clause et al., 2017), suppression of auditory distractors during selective attention to visual stimuli (Terreros et al., 2016), vestibular rehabilitation (Eron et al., 2015), motion sickness and balance control (Tu et al., 2017), stress responses (Colomer et al., 2010; Mohammadi et al., 2017), awake/sleep cycle and circadian rhythm regulation (Velluti et al., 1989; Madrid-López et al., 2017; Mohammadi et al., 2017), and modulation of pain and hyperalgesia (Mohammadi and Christie, 2014; Romero et al., 2017).

Selective attention is the capacity to focus cognitive resources on a relevant stimulus while suppressing irrelevant stimuli. It is proposed that selective attention activates high order cortical regions, while filtering peripheral responses in a top-down manner, allowing subjects to maintain goal-directed behaviors in the presence of distractors (Fritz et al., 2007). In addition to the behavioral response oriented to the attended stimulus, inhibition of the behavioral responses to irrelevant stimuli is an important mechanism of the selective attention processes (Dalley et al., 2008). In this line the auditory efferent system, including OC neurons are important pathways that permit filtering of cochlear and auditory nerve responses during selective attention to visual stimuli (Delano et al., 2007; Smith et al., 2012).

The different behavioral errors produced during a cued attention task can be classified as premature, target and perseverative errors (Muir, 1996). Premature errors are reflected by responses to cue stimulus, and could be seen as a measure of impulsivity, while target errors (incorrect responses) measure the decision-making process, and perseverative errors are indicators of loss of inhibitory control. In this sense, perseverative responses can be considered as a possible index of compulsive action or an inability to inhibit the repetition of an action already planned, while premature responses can be considered as an index of the ability to wait or retain an action (Eagle and Baunez, 2010). Moreover, error responses are used as behavioral indicators related to attention disorders such as attention deficit hyperactivity disorder (Malloy-Diniz et al., 2007; Eagle and Baunez, 2010; Nandagopal et al., 2011; Lopez et al., 2015), and other neuropsychiatric conditions related with frontal-basal ganglia network dysregulation like Tourette syndrome, obsessive compulsive disorder and frontotemporal dementia and Alzheimer’s disease (Jahanshani and Rothwell, 2017). Premature and perseverative actions are related to dysfunction of different anatomical or neurochemical mechanisms (Evenden, 1999). For example, premature responses with short latencies, measured in the five-choice serial reaction time increase in rats with infra-limbic lesions, while rats with orbitofrontal lesions produce perseverative responses in the same behavioral paradigm (Chudasama et al., 2003). In this line, attentional markers respond to complex neurobiological pathways, including prefrontal, limbic and striatal regions (Urcelay and Dalley, 2012).

Recently, we published evidence that an intact cholinergic MOC transmission aids in ignoring auditory stimuli during selective attention to visual stimuli (Terreros et al., 2016). In that work, we used α9 nicotinic receptor subunit (α9-nAChR) knock-out (KO) mice, which lack cholinergic transmission between MOC and OHC (Vetter et al., 1999), and found that α9-nAChR KO mice made more omissions and fewer correct responses than wild type (WT) mice in a visual selective attention task with auditory distractors. However, whether the lack of α9-nACh subunit receptors produces consequences in the neural network of behavioral errors is unknown. Here, we studied premature, target and perseverative errors during visual selective attention with auditory distractors in WT and α9-nAChR KO mice. We found that α9-nAChR KO mice make fewer perseverative errors with longer latencies than WT mice.

## Materials and Methods

All procedures were approved by the local committee of Bioethics (Comité de Bioética Animal permit number #0548 Facultad de Medicina, Universidad de Chile) and were performed in accordance with the Guidelines for the Care and Use of Laboratory Animals (publication number 86–23, National Institutes of Health, revised 1996). Efforts were made to minimize the number of animals used and their suffering.

### Animals

We analyzed data from the same group of animals that were used for the work published in Terreros et al. (2016), corresponding to 15 WT and 17 α9-nAChR KO male mice aged between 60 days and 80 days at the start of behavioral training. Perseverative errors, total lever responses and latencies to error responses presented in this article were not included in Terreros et al. (2016). KO mice on the 129/SvEv backcrossed to CBA/CaJ background (Vetter et al., 1999) and WT littermates were generously provided by Dr. Douglas Vetter from the University of Mississippi. Deletion of α9 nicotinic acetylcholine receptor subunit was confirmed for each mouse by PCR screening of genomic DNA extracted and purified from the tail. Mice were housed in groups of two in temperature-controlled conditions (22°C ± 2°C) with a 12-h dark/light cycle (lights off at 8.00 A.M) with *ad libitum* access to water. They were food deprived during the experimental period, maintaining 84%–92% of their free-feeding weight. Detailed results from accuracy, correct responses, and omissions during the behavioral protocol of these mice and from auditory brainstem responses (ABR) can be found in Terreros et al. (2016).

### Training Procedures

Visual attention was assessed in a two-choice visual discrimination task, based on that we used previously in rats (Hamame et al., 2006, 2008), chinchillas (Delano et al., 2007) and mice (Terreros et al., 2016). The operant mesh cage (17 cm long, 20 cm wide and 28 cm high) was located inside a double-walled sound-attenuating room. The front panel of the cage had two lateral lights above two levers and a central light (neutral cue). Each trial began with the onset of the neutral cue for 2 s, followed by the random turn on of one of the two lateral lights for 0.5 s. Mice were trained to press the lever located below the target light during the response period, 5 s from the onset of the target light. Correct responses were rewarded with a 15-mg precision pellet (Bioserv^®^) through a food dispenser magazine. Lever pressing opposite to the target light during the response period was defined as an incorrect response (target error). The intertrial interval (ITI) period varied randomly between 5 s and 11 s. Responses to the central light (cue) were considered as premature responses. Incorrect responses or any lever press during the central light and ITI was punished with a 12 s period during which all lights were turned off. If mice pressed the lever during this punish time, the 12 s period was reinitiated and was considered as a perseverative response. Omissions occurred when mice did not respond to the neutral cue or target lights, and was not punished. The behavioral variables measured were accurate ([correct responses/(correct responses + incorrect responses)] * 100), correct responses, incorrect responses, number of omitted trials, perseverative responses, number of responses during the central light (premature responses) and ITI periods. Non-target errors were considered as the sum of premature, perseverative and ITI responses. Responses latencies: premature response latency represents the time between the onset of the central light and a lever pressing, incorrect response latency represents the time between the onset of the target light and a lever pressing opposite to it, ITI responses latency represents the time between the end of the response period and a lever pressing, and perseverative response latency represents the time between the start of the punish time and a lever pressing.

### Experimental Protocol

After mice reached an accuracy of at least 70% for three consecutive days, they were recruited in the experimental protocol. The 12 days of experimental protocol was divided into four periods of 3 days with 110 trials each day. In the first 3 days (pre-distractor period, PRE), mice performed the visual discrimination task without auditory distractors. During days 4–6, mice did the visual discrimination task in the presence of clicks and 15 kHz tones (C+T) as auditory distractors and during days 7–9 in presence of broadband noise (BBN). Finally, in the last 3 days of the experimental protocol (days 10–12, post-distractor period, POST), mice were again evaluated in the visual discrimination task without auditory distractors.

### Auditory Distractors

The C+T auditory distractor consisted of a click (100 μs wide) followed by a 15 kHz tone presented in every trial 40 ms after the click at ~67 dB SPL. BBN auditory distractors (5–40 kHz) were presented at ~90 dB SPL. All acoustic stimuli were digitally generated at 100 kHz with a National Instruments Board (PC-MIO-16E4). Tones and BBN had a 5 ms ramp (rise/fall) and a total duration of 150 ms. To diminish habituation to auditory distractors, auditory stimuli were delivered at irregular inter-stimulus intervals that were centered at 400 ms (2.5 Hz rate, 1.5–3.5 Hz rate, uniformly distributed intervals) and pseudorandomly ranged between 667 ms and 286 ms. Clicks, tones, and BBN were delivered through a tweeter (Realistic super tweeter; Radioshack; frequency response 5–40 kHz) located 120 cm above the floor of the operant cage. Sound pressure calibrations inside the operant cage were performed with a 1/2″ Bruel and Kjaer^®^ microphone that revealed <10 dB of variation in different positions of the behavioral cage.

### Electrophysiological Procedures

The presence of wave I from ABR was used to obtain auditory thresholds using ipsilateral 15 kHz tone bursts at different sound pressure levels (from 0 dB to 90 dB SPL). ABR thresholds were defined as the lowest tone intensity (dB SPL) that evoked an averaged response evaluated by visual inspection of wave I by an expert observer. In addition, the amplitudes and latencies of ABR waves (I-V) in response to 15 kHz tones at 80 dB were measured in both genotypes.

### Statistical Analyses

Behavioral data were analyzed by two-way repeated-measures analysis of variance (RM-ANOVA) and were followed by Bonferroni *post hoc* comparison tests. Non-normal distributed data were transformed to [Log (X + 1)] to satisfy requirements of the ANOVA model. Difference in the distribution of behavioral latencies was evaluated by Kolmogorov-Smirnov test. Differences in ABR amplitudes, latencies and thresholds were evaluated by *t*-tests. Significant differences were considered for statistical tests with *p-value* <0.05.

## Results

**Figure 1:**
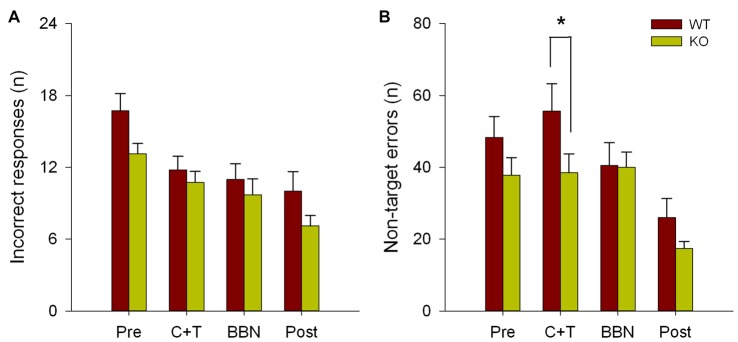
Difference in the number of non-target errors between wild type (WT) and knock-out (KO) mice during the C+T period. Incorrect and non-target responses are shown in red and green bars (mean ± SEM) for WT and KO mice correspondingly. **(A)** Number of incorrect responses. **(B)** Number of non-target errors. *p < 0.05.

**Figure 2:**
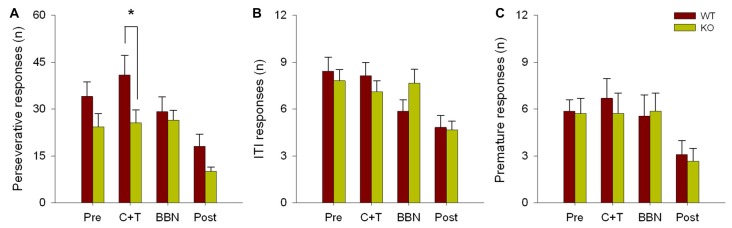
Difference in the number of perseverative responses between WT and KO mice during the C+T period. The different behavioral measures in the four periods of the experimental protocol are shown in red and green bars (mean ± SEM) for WT and KO mice correspondingly. **(A)** Number of perseverative responses. **(B)** Number of intertrial interval (ITI) responses. **(C)** Number of premature responses. *p < 0.05.

The number and mean latencies of incorrect and non-target error responses (premature, perseverative and ITI responses) in WT and KO mice were compared during the four periods of the experimental protocol (PRE, C+T, BBN and POST). Figure 1 shows that there were no differences in the number of incorrect responses between WT and KO mice, while a significant difference was found in non-target error responses (two way RM-ANOVA, *F*_(1,3)_ = 12.374, *p* < 0.001). Bonferroni *post hoc* tests showed that during the C+T period KO mice had significantly fewer non-target error responses (*t* = 2.302, *p* < 0.05; WT = 55.689 ± 29.517 (mean ± SD), KO = 38.371 ± 21.841). As non-target error responses considered the sum of premature, perseverative and ITI responses, we separated behavioral data into these three possible error responses. Figure 2 shows that there was a significant difference in perseverative responses (Two way RM-ANOVA, *F*_(1,3)_ = 10.683, *p* < 0.001; WT = 40.867 ± 24.475, KO = 25.627 ± 16.701) during the C+T period between WT and KO mice by a Bonferroni *post hoc* test (*t* = 2.588, *p* < 0.05), while non-significant differences were found in ITI and premature responses between both genotypes.

**Figure 3:**
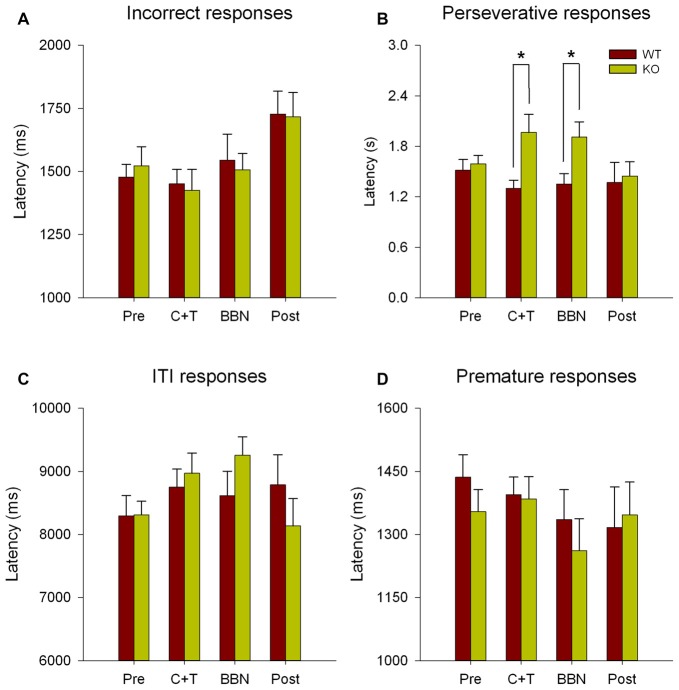
Differences in the mean latency of perseverative responses between WT and KO mice during C+T and broadband noise (BBN) periods. The latencies of error responses in the four periods of the behavioral protocol are shown in red and green bars (mean ± SEM) for WT and KO mice correspondingly. **(A)** Mean latencies of incorrect responses. **(B)** Mean latencies of perseverative responses. **(C)** Mean latencies of ITI responses. **(D)** Mean latencies of premature responses. *p < 0.05.

Next, we compared the latency of all error responses, including incorrect, premature, perseverative and ITI responses. Figure 3 shows significant differences in the latencies of perseverative responses (Two-way RM-ANOVA, *F*_(1,3)_ = 2.850, *p* < 0.05) between WT and KO. Bonferroni *post hoc* tests show significant decreases in the mean latency of perseverative responses in WT mice during C+T (*t* = 2.834, *p* < 0.01; WT = 1.300 ± 0.378 s, KO = 1.965 ± 0.891 s) and BBN periods (*t* = 2.374, *p* < 0.05; WT = 1.352 ± 0.483 s, KO = 1.908 ± 0.750 s). No differences were found in the latencies of incorrect, premature and ITI responses.

**Figure 4:**
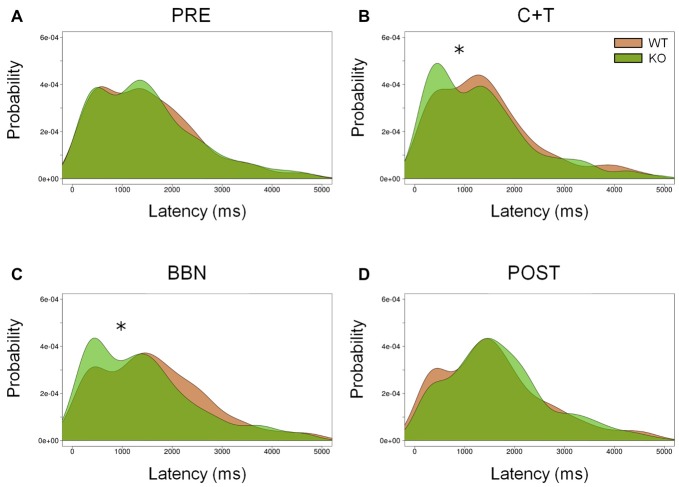
Differential distribution of incorrect responses latencies between WT and KO mice during C+T and BBN periods. Density of incorrect responses latencies in the four periods of the behavioral protocol are shown in red and green lines for WT and KO mice correspondingly. Notice the presence of two peaks, around 500 ms and 1500 ms in the four periods. KO mice increase the amplitude of the first peak of incorrect responses with auditory distractors (C+T and BBN periods). **(A)** Pre-distractor period. **(B)** C+T period. **(C)** BBN period. **(D)** Post-distractor period. Kolmogorov-Smirnov test, *p < 0.05.

In addition, we analyzed the distribution of error latencies between WT and KO mice in the four experimental periods. Distributions of premature and ITI responses were similar between genotypes in the four experimental periods. Figure 4 shows the presence of two latency peaks of incorrect responses in the four periods in both genotypes, the first peak is around 500 ms, while the second is near 1500 ms. We found a significant increase of the first latency peak of incorrect responses in KO mice compared to WT mice during the presence of auditory distractors (C+T and BBN periods; Kolmogorov-Smirnov test (C+T, *D* = 0.093, *p* < 0.05; BBN, *D* = 0.119, *p* < 0.01). Regarding perseverative responses, there were significant differences in the latency distribution between genotypes during the four periods (PRE, *D* = 0.092, *p* < 0.005; C+T, *D* = 0.124, *p* < 0.005; BBN, *D* = 0.070, *p* < 0.005; POST, *D* = 0.126, *p* < 0.005), showing a decrease in the latency peak of perseverative responses in WT mice.

**Figure 5:**
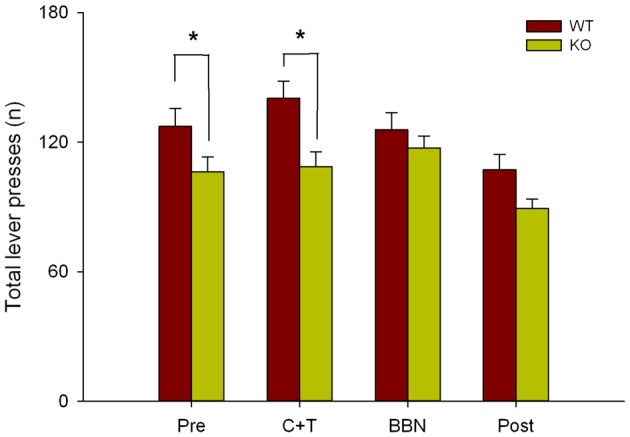
Differences in the total number of lever presses between WT and KO mice during PRE and C+T periods. The number of total lever presses in the four periods of the behavioral protocol are shown in red and green bars (mean ± SEM) for WT and KO mice correspondingly. *p < 0.05.

Next, as in the present study we found that in the presence of auditory distractors WT mice made more perseverative errors, and in our previous work we showed that WT mice made more correct responses and less omissions than KO mice (Terreros et al., 2016), we compared the total number of lever presses between genotypes in the four experimental periods. Figure 5 shows a significant difference in the total number of lever presses between both genotypes (Two-way RM-ANOVA, *F*_(1,3)_ = 10.143, *p* < 0.001) during PRE (Bonferroni *post hoc, t* = 2.170, *p* < 0.05; WT = 127.333 ± 32.571, KO = 106.294 ± 28.282) and C+T periods (*t* = 3.260, *p* < 0.05; WT = 140.378 ± 30.529, KO = 108.765 ± 28.123), showing that WT mice made more lever presses than KO mice.

Finally, as previous evidence in α9-KO mice showed an altered development of the responses of brainstem auditory neurons (Clause et al., 2014); we analyzed ABR at thresholds and supra-thresholds levels in both genotypes. We found non-significant differences in wave I thresholds between genotypes (WT = 31.0 ± 4.7 dB; KO = 31.9 ± 4.4 dB, *t*-test), and non-significant differences in the supra-thresholds amplitudes of wave I and V (wave I: WT = 0.46 ± 0.12 μV, KO = 0.49 ± 0.10 μV; wave V: WT = 0.50 ± 0.12 μV, KO = 0.52 ± 0.11 μV, *t*-tests). Regarding ABR latencies, there were no genotype differences in waves I, II and III (wave I_(WT)_ = 1.76 ± 0.45 ms; wave I_(KO)_ = 1.80 ± 0.37 ms; wave II_(WT)_ = 3.44 ± 0.33 ms; wave II_(KO)_ = 3.44 ± 0.36; wave III_(WT)_ = 4.60 ± 0.31 ms; wave III_(KO)_ = 4.61 ± 0.33), while we found a significant difference between genotypes in the latency of wave V (WT = 6.60 ± 0.37 ms; KO = 6.88 ± 0.33 ms, *t*-test *p* < 0.05).

## Discussion

We show that WT mice made more perseverative errors with shorter latencies than α9-nAChR KO mice during selective attention to visual stimuli with auditory distractors. In addition, while we found no significant difference in the number of incorrect responses between genotypes, KO mice increased the frequency of short-latency incorrect responses during the presentation of the click and tone distractors. These behavioral differences should be taken together with our previous results of the same group of mice (Terreros et al., 2016), in which KO mice omitted more trials and made fewer correct responses than WT mice in the presence of click and tone distractors.

### Difference in the Distribution of Incorrect Responses with Auditory Distractors

Although, there were no differences in the number of incorrect responses with auditory distractors between genotypes, we found a differential distribution of incorrect responses during the presentation of auditory distractors, reflected by the increase of the short-latency peak of incorrect responses in KO mice (around 500 ms). The significant increase of early incorrect responses could be indicative of more impulsive decision in KO mice induced by auditory distraction in a visual selective attention task. Auditory-cortex descending pathways can be though as a neural circuit that allows top-down filtering of afferent auditory responses (Xiao and Suga, 2002; León et al., 2012; Aedo et al., 2016). In that line, we propose that the increased auditory distraction in KO mice could be consequence of the lack of OHC inhibition by MOC synapses in the KO mice. This might impair the filtering of auditory distractors by the corticofugal projections from the auditory cortex that modulate MOC neurons (Dragicevic et al., 2015; Aedo et al., 2016). Importantly, auditory thresholds and supra-thresholds ABR amplitudes were similar for WT and KO mice, and only a significant difference in the latency of wave V was obtained. With these results, we suggest that afferent responses to auditory distractors were relatively similar in both genotypes, and that the lack of top-down filtering of irrelevant auditory distractors in KO mice might be the principal contributor to impulsive behaviors during decision making with auditory distractors.

### Difference in Perseverative Errors

WT mice made more perseverative errors with shorter latencies than KO mice during visual selective attention with auditory distractors. These unexpected results are probably not related to the lack of functional MOC synapses in the cochlear receptor of KO mice, and could be attributed to the expression of α9-nAChR in tissues different than the inner ear. In the nervous system, besides cochlear hair cells (Elgoyhen et al., 1994), the α9 subunit of the nicotinic receptor has been found in vestibular hair cells (Hiel et al., 1996; Luo et al., 1998), dorsal root ganglia (Lips et al., 2002), and retina (Smith et al., 2014), while in the endocrine system has been detected in the pituitary and adrenal glands (Elgoyhen et al., 1994; Colomer et al., 2010). Colomer et al. (2010) found that α9-nACh receptors are upregulated in response to cold stressors, while Mohammadi et al. (2017) evidenced that α9-nAChR KO mice have dysregulated stress responses, increasing plasma corticosterone levels in α9-nAChR KO compared to WT. In line with this explanation, our results could be related to the effects of stress hormones on the medial prefrontal cortex (mPFC). Therefore, it is possible that training in the attentional task was a chronic mild stress for the α9-nAChR KO mice. In this context, chronic stress alters mice mPFC, a brain area involved on inhibitory control (Gilabert-Juan et al., 2013; Agoglia et al., 2017). Together, these findings suggest that in our experiments training stress might have deteriorated the mPFC of the α9-nAChR KO. Moreover, this idea is supported by a recent work showing the expression of alpha-9 nicotinic receptors in the frontal cortex, basal ganglia and thalamus of mice that might explain the difference in perseverative errors observed in KO mice (Lykhmus et al., 2017). The presence of alpha-9 receptors in these brain regions could also affect the neural circuits of motivation and reward, explaining fewer perseverative errors and fewer total lever responses observed in the α9-nAChR KO mice. Mohammadi et al. (2017) showed that α9-nAChR KO mice displayed an anhedonia-like behavior in a sucrose preference test, while WT mice continued to seek for reward. The higher number of perseverative errors that we found in the WT mice could be related with the instrumental learning paradigm used in our study, which is solely based on a reward outcome, without any aversive punishment. These types of paradigms produce a robust “action” or “go for reward” response in normal subjects (Guitart-Masip et al., 2012). In this line, the motivation to seek for a reward could also explain the significantly larger amount of total lever presses observed in the WT in comparison with the KO mice in the first two periods (PRE and C+T) of the experimental protocol (Figure 5). A reduced motivation in α9-nAChR KO mice is also supported by our previous work (Terreros et al., 2016), in which we found that WT mice made more correct responses and fewer omissions than KO mice without changes in locomotor activity as measured through open field test. In this line, correct responses and perseverative errors could be indicative of a motivated behavior, in which WT mice are seeking for reward, and consequently, considering that both genotypes had a similar level of food deprivation (no weight differences), fewer total lever responses in KO mice could reflect less motivation (Jones et al., 2017). In complement with the previous idea, at the 4th day of the experimental protocol when mice have completed their learning phase, they probably have changed their responses from an “action-outcome learning” to a “stimulus response learning” (Yin and Knowlton, 2006). In the latter type of behavior is frequent to see generalization of responses (go) with different stimulus, presuming that the WT generalizes the response from a visual to an auditory trigger (Jones et al., 2017), this hypothesis could explain why these types of errors are overexpressed in the C+T period.

## Conclusion

Here we demonstrated that perseverative errors are diminished in α9-nAChR KO mice compared to WT mice during visual selective attention with auditory distractors, suggesting a reduced motivation for reward in KO mice. In addition, we found that in the presence of auditory distractors, the early latency peak of incorrect responses is increased in α9-nAChR KO mice, suggesting that the lack of MOC function increases impulsivity during decision making with auditory distraction.

## Author Contributions

PJ, GT and PHD designed research; PJ and GT performed research; PJ analyzed the data; PJ, GT, PHD, AD-S and CD contribuited and wrote the article. PHD, CD and AD-S contributed with analytic tools and unpublished reagents.

## Conflict of Interest Statement

The authors declare that the research was conducted in the absence of any commercial or financial relationships that could be construed as a potential conflict of interest.
